# Diagnostic value of percutaneous paramedian small-angle lateral intervertebral foramen Kambin’s triangle approach for lumbar puncture biopsy combined with tNGS-based multimodal etiological diagnosis in early spinal infection: a multicenter retrospective diagnostic yield study

**DOI:** 10.3389/fcimb.2026.1865471

**Published:** 2026-07-15

**Authors:** Yuxin Gao, Linan Wang, Jiong Wang, Xingyu Duan, Hongbao Ma, Zhiwei Ma, Jiaxing Wang, Zhiyun Shi, Ningkui Niu

**Affiliations:** 1 Department of Orthopedics, General Hospital of Ningxia Medical University, Yinchuan, China; 2 The First Clinical Medical College of Ningxia Medical University, Yinchuan, China; 3 Department of Orthopedics, The People’s Hospital of Yongning County, Yinchuan, China; 4 Department of Orthopedics, The Third People’s Hospital of Yinchuan, Yinchuan, China; 5 Medical Experiment Center, General Hospital of Ningxia Medical University, Yinchuan, China; 6 Research Center for Prevention and Control of Bone and Joint Tuberculosis, General Hospital of Ningxia Medical University, Yinchuan, China

**Keywords:** early diagnosis, etiological diagnosis, intervertebral disc puncture, spinal infection, targeted next-generation sequencing (tNGS)

## Abstract

**Objective:**

To validate the safety and multicenter technical feasibility of an innovatively designed percutaneous paramedian small-angle lateral intervertebral foramen Kambin’s triangle approach for lumbar puncture biopsy in early spinal infection (SI), and to evaluate the diagnostic yield and etiological identification rate of the tNGS-based multimodal etiological detection protocol using specimens obtained via this approach.

**Methods:**

This was a multicenter retrospective cohort study that enrolled 94 consecutive patients with SI who underwent the aforementioned puncture biopsy at 3 medical centers between January 2024 and January 2026. We systematically evaluated the procedure-related indicators and perioperative complications of this puncture approach. All puncture specimens were synchronously subjected to histopathological examination, routine bacterial culture, Xpert MTB/RIF assay, and tNGS detection. With the final comprehensive clinical diagnosis as the reference standard, we compared and analyzed the diagnostic performance of each detection method.

**Results:**

All 94 patients successfully completed biopsy via this modified puncture approach, with an overall puncture-related complication rate of only 7.45%. All adverse events were mild, and no severe complications occurred, and the approach was feasible and appeared safe in this retrospective cohort, though prospective validation across multiple operators is warranted before widespread clinical adoption. For etiological detection, the overall positive rate of tNGS reached 87.23%, which was significantly higher than that of routine bacterial culture (25.53%), specific histopathological diagnosis (17.02%), and Xpert MTB/RIF assay (17.02%) (all *P* < 0.0001). Meanwhile, tNGS maintained a stable and high detection rate across all major SI subtypes. The tNGS-centered multimodal combined detection achieved an overall positive rate of 91.49%. Notably, in early-stage cases where histopathology only indicated non-specific inflammatory cell infiltration, the combined detection achieved an etiology confirmation rate of 95.65%, effectively compensating for the deficiency of detection methods in early diagnosis.

**Conclusions:**

The puncture approach designed in this study addresses the core bottleneck of specimen acquisition in early SI. The tNGS-based multimodal detection system using this approach has excellent diagnostic performance in early-stage infection, and can provide critical evidence-based support for the early accurate diagnosis and treatment as well as targeted antimicrobial therapy of SI.

## 1 Introduction

Spinal infection (SI) is a group of infectious disorders involving the vertebral bodies, intervertebral discs, and paravertebral soft tissues, with spinal tuberculosis (STB), brucellar spondylitis (BS), and pyogenic spondylitis (PS) as the three major subtypes. It has a reported global annual incidence of 1.8 to 7.0 per 100, 000 population (Henry et al., 2025; Yu et al., 2025). Early SI lacks specific clinical manifestations and radiological features, which frequently results in delayed diagnosis and management and subsequent devastating adverse outcomes including paraplegia and septic shock. Thus, establishing a safe, efficient, and accurate early diagnostic system is a core priority for improving clinical outcomes in affected patients (Shroyer et al., 2022; Zou et al., 2025).

Histopathological examination has historically been regarded as one of the reference gold standards for SI diagnosis, which relies on the surgical acquisition of lesion specimens. However, most patients with early-stage disease have no indications for open surgery, resulting in a restricted window for specimen acquisition. Furthermore, the majority of early lesions present only with non-specific inflammatory cell infiltration, while specific pathological features are observed only in a small number of patients with intermediate to advanced tuberculosis. This renders histopathology unable to provide critical information for targeted therapy, such as the identification of pathogenic species (Bürger et al., 2020; Xu et al., 2026). In recent years, molecular diagnostic technologies centered on next-generation sequencing (NGS) have provided novel strategies for SI diagnosis and management. Among these, metagenomic NGS (mNGS) has broken through the limitations of routine bacterial culture via its broad-spectrum coverage of all pathogens and excellent diagnostic sensitivity; however, its high cost and complex workflow have hindered its widespread routine clinical application (Han et al., 2019). In contrast, targeted NGS (tNGS) is more aligned with the clinical needs of early SI diagnosis and management. It can specifically cover hundreds of pathogens associated with orthopedic infections. Chang et al. (Yang et al., 2025) demonstrated in the diagnosis of urinary tract infections that the turnaround time of tNGS was only 12.89 hours, significantly shorter than that of routine bacterial culture (61.48 hours) and mNGS. It also achieved a higher detection rate in culture-negative samples (53.1%) than mNGS (28.1%), with a 55.4% detection rate for polymicrobial infections. While maintaining high sensitivity, tNGS also substantially reduces testing costs and shortens reporting turnaround time. Molecular diagnostic technologies represented by tNGS have drastically improved the diagnostic performance for early etiological identification, and have also sparked a core debate in the SI field: whether the early SI diagnostic system should be repositioned to elevate multimodal etiological diagnosis to a central role, while redefining histopathological examination as an auxiliary tool for differential diagnosis (Fransen et al., 2014; Xu et al., 2026).

Regardless of how the diagnostic system is positioned, qualified lesion specimens remain an essential prerequisite. As most patients with early SI have no indications for open surgery, minimally invasive percutaneous puncture biopsy of the infected intervertebral disc has become the preferred method for specimen acquisition. The posterolateral far-lateral approach, the most widely used puncture method in current clinical practice, requires a long puncture trajectory. This approach is prone to cause injury to the paraspinal muscles, psoas major muscle, and paravertebral vessels, as well as postoperative chronic low back pain. Furthermore, it requires a large medial angulation to reach the target area, and operational deviations can easily lead to complications such as dural tear and nerve injury, which limit its widespread clinical application (Tang et al., 2025).

To address these clinical limitations, we developed an innovative percutaneous paramedian small-angle lateral intervertebral foramen Kambin’s triangle approach for lumbar puncture biopsy. This technique leverages the anatomical safe window of the spine to shorten the puncture trajectory and enable the acquisition of qualified specimens with minimal trauma and high procedural stability. We simultaneously established a multimodal etiological detection protocol combined with tNGS, and conducted a retrospective diagnostic yield study enrolling 94 patients with SI from 3 medical centers. The aims of this study were to systematically validate the safety and feasibility of this innovative puncture approach as a reliable specimen acquisition platform for early SI, and subsequently to clarify the diagnostic value of the tNGS-based multimodal etiological detection protocol using specimens obtained via this approach for early etiological confirmation, ultimately provide high-quality evidence-based medical evidence for the optimization of early SI diagnosis and management strategies.

## 2 Materials and methods

### 2.1 Study design and ethical approval

**Figure 1:**
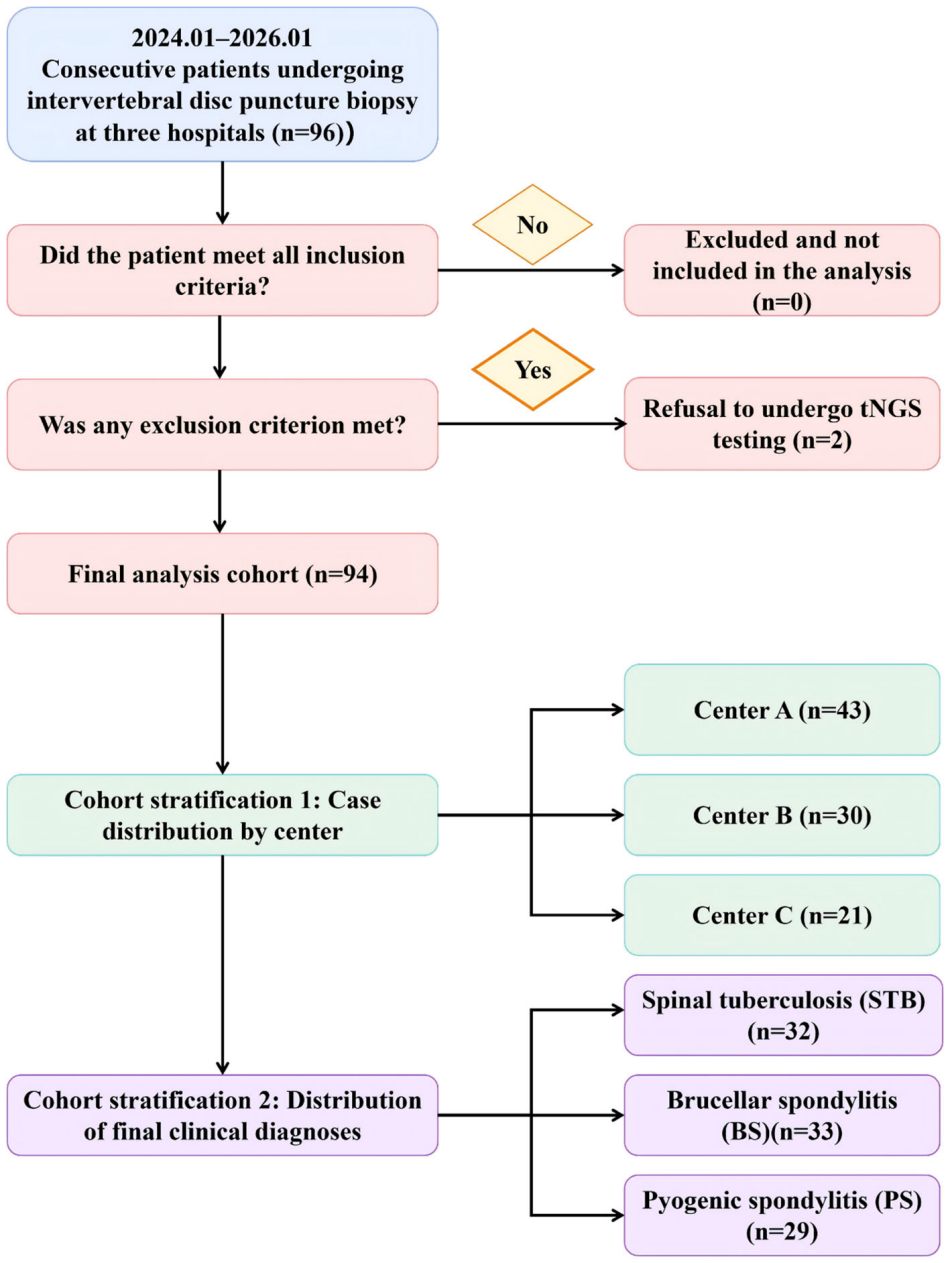
Flow diagram of patient cohort selection.

This was a multicenter retrospective diagnostic yield study conducted in strict accordance with the ethical principles of the Declaration of Helsinki and the Strengthening the Standards for Reporting Diagnostic Accuracy Studies (STARD).The patient screening flowchart is presented in **Figure 1**. Consecutive patients with spinal infection who underwent the aforementioned percutaneous puncture biopsy at 3 participating medical centers between January 2024 and January 2026 were retrospectively enrolled in this study. All procedures were performed independently by a single senior attending surgeon at each center after completion of standardized training. The study design followed a single-group diagnostic yield framework: all enrolled patients underwent the index diagnostic strategy (tNGS-based multimodal etiological detection) and the reference standard (final comprehensive clinical diagnosis), enabling direct comparison of the index test against the reference standard. All enrolled patients underwent the identical diagnostic workup; no patient grouping by exposure factor or treatment allocation was performed.

The study protocol was approved by the Institutional Review Board of the leading center, General Hospital of Ningxia Medical University (approval number: KYLL-2025-1127). This approval was formally acknowledged and filed by the IRBs of the other two participating centers. Written informed consent for the puncture and diagnostic procedure was obtained from all patients prior to the operation. The retrospective analysis of anonymized patient data was approved by the IRB with a waiver of additional informed consent.

### 2.2 Study population

#### 2.2.1 Inclusion criteria

Consecutive patients were included in this study if they met all of the following criteria: preoperative high suspicion of SI based on clinical symptoms, inflammatory biomarkers, and imaging examinations; no indications for open surgery for SI and no prior relevant open surgery before enrollment; successful completion of the specified percutaneous lumbar puncture biopsy in this study with qualified lesion specimens acquired; and complete clinical diagnosis and treatment data available for the final comprehensive clinical diagnosis.

#### 2.2.2 Exclusion criteria

Patients were excluded from this study if they met any of the following criteria: had undergone emergency open surgery for SI; had absolute contraindications to percutaneous puncture biopsy; were deemed ineligible for the anatomical application of the study puncture approach based on preoperative imaging assessment; received a final comprehensive clinical diagnosis of non-infectious spinal disorders; or had incomplete clinical data that precluded statistical analysis of core indicators.

#### 2.2.3 Definition of early-stage spinal infection

In this study, early-stage SI was defined by the following clinical and radiological criteria: (1) imaging (MRI/CT) showing disc-space involvement and endplate erosion without extensive vertebral body destruction or significant spinal deformity; (2) no extensive paravertebral or epidural abscess requiring open surgical drainage; (3) no neurological deficit attributable to spinal cord or cauda equina compression; and (4) no prior open surgery for SI. All 94 enrolled patients met these criteria.

### 2.3 Data collection and variable definition

Standardized data collection and verification were performed by research personnel who had completed unified standardized training, using the electronic medical record system of each participating center. The collected data included demographic characteristics, baseline clinical data, preoperative inflammatory biomarkers and serological test results, imaging data, puncture procedure-related indicators, as well as etiological test results including histopathology, tNGS, routine bacterial culture, and Xpert MTB/RIF assay.

### 2.4 Operative specification of percutaneous paramedian small-angle lateral intervertebral foramen Kambin’s triangle approach for lumbar puncture biopsy

#### 2.4.1 Surface localization and anesthesia

**Figure 2:**
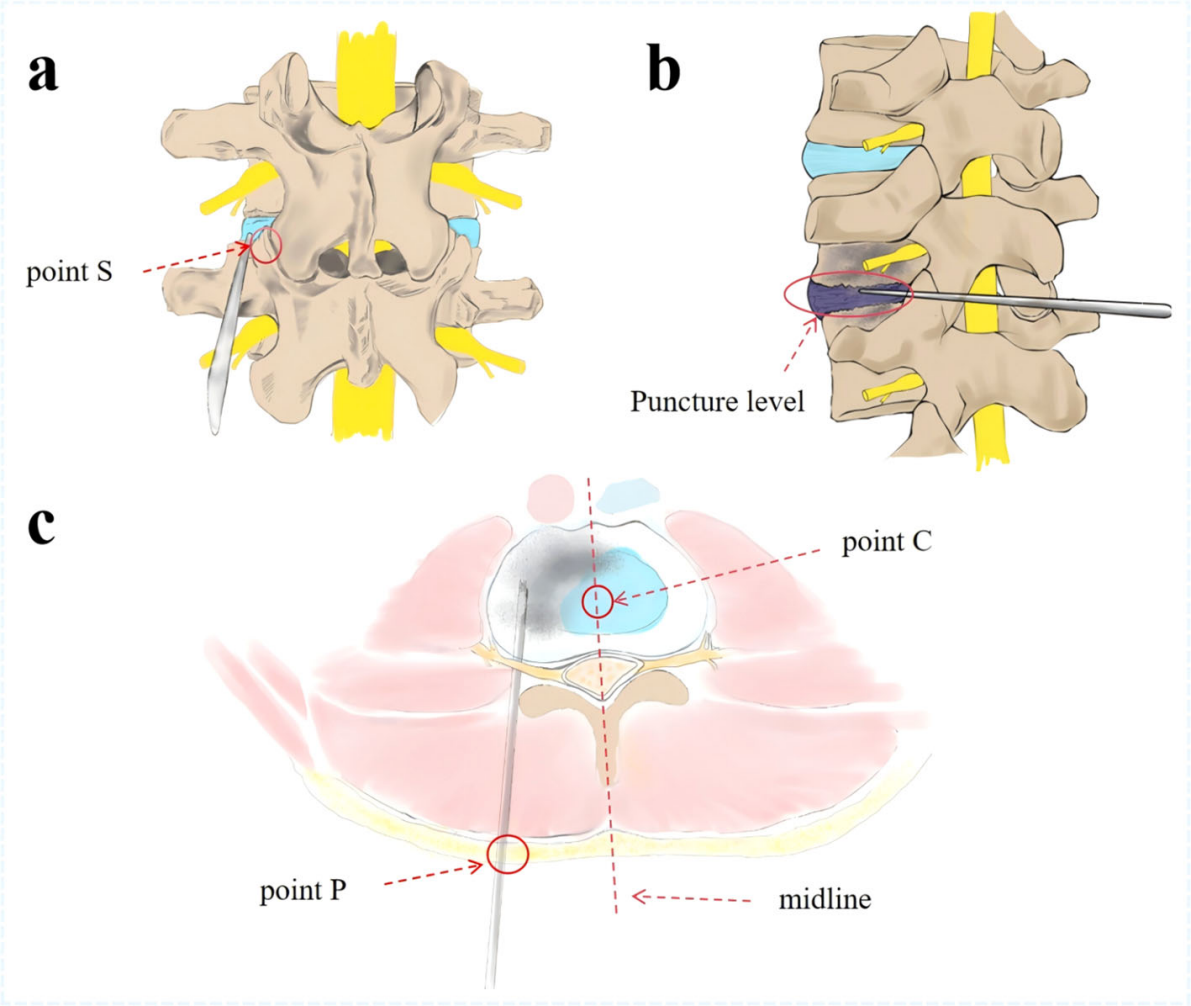
Schematic anatomical diagram of the puncture procedure. Panel a shows the schematic anatomical diagram of coronal puncture in the patient; Panel b shows the schematic anatomical diagram of 45° sagittal puncture in the patient; Panel c shows the schematic anatomical diagram of axial puncture in the patient.

Patients were placed in the prone position, and the entire procedure was performed under C-arm fluoroscopy guidance. A 22G sharp-tipped guide needle, 18G/16G hollow puncture biopsy needles, and matched biopsy instruments were prepared in advance. Anteroposterior fluoroscopy was performed to mark the projection point of the target intervertebral disc center (point C). With the spinous process of the target segment as the midline, the skin entry point (point P) was marked with a lateral offset of 1–2 cm (baseline: 1.5 cm) from the midline on the symptomatic side (the side with more severe lesions; the left side was selected for bilateral lesions to avoid the inferior vena cava), ensuring that point P and point C were at the same horizontal axial level. For obese patients, point P could be laterally adjusted by 0.2–0.5 cm under fluoroscopic guidance. Routine skin disinfection and surgical draping were performed with point P as the center, and a mixture of lidocaine and ropivacaine was administered for layered infiltration anesthesia from the skin to the periosteum at the posterolateral edge of the vertebral body. The specific puncture procedure and anatomical localization are shown in **Figure 2**.

#### 2.4.2 Core puncture procedure steps

##### 2.4.2.1 Initial guide needle insertion and establishment of the safety pivot point

The guide needle was inserted vertically from point P. After penetrating the deep fascia, it was adjusted with an amplitude of ≤5° under AP fluoroscopy and advanced toward the posterolateral corner of the target intervertebral disc (the posterior boundary of Kambin’s triangle), until it touched the lateral edge of the superior articular process of the inferior vertebral body and bone resistance was felt. This point was defined as the safety pivot point (point S).

##### 2.4.2.2 Gliding insertion of the guide needle and initial target localization

Using point S as the pivot, the needle tip was kept in close contact with the bone surface and glided slowly in an anteromedial direction (toward the center of the intervertebral disc) with a medial inclination angle of ≤8°. Needle insertion was paused when the tip entered the lateral region of the intervertebral space.

##### 2.4.2.3 Biplane fluoroscopic verification of guide needle target position

Lateral fluoroscopy was switched to verify that the needle tip was anterior to the posterior edge of the vertebral body and within the posterior 1/3 of the intervertebral space. Insertion was continued if the position was satisfactory; for suboptimal positioning, the needle was adjusted with an amplitude of ≤3°followed by repeated fluoroscopic verification. This process was repeated until the guide needle penetrated the annulus fibrosus (with a palpable give-way sensation). Lateral fluoroscopy confirmed that the needle tip was located in the target nucleus pulposus area in the middle and posterior 1/3 of the intervertebral space, and AP fluoroscopy confirmed its position in the central area of the intervertebral disc, completing guide needle localization. The total medial inclination angle was ≤30° throughout the entire procedure.

##### 2.4.2.4 Coaxial insertion of the hollow puncture needle and final position confirmation

**Figure 3:**
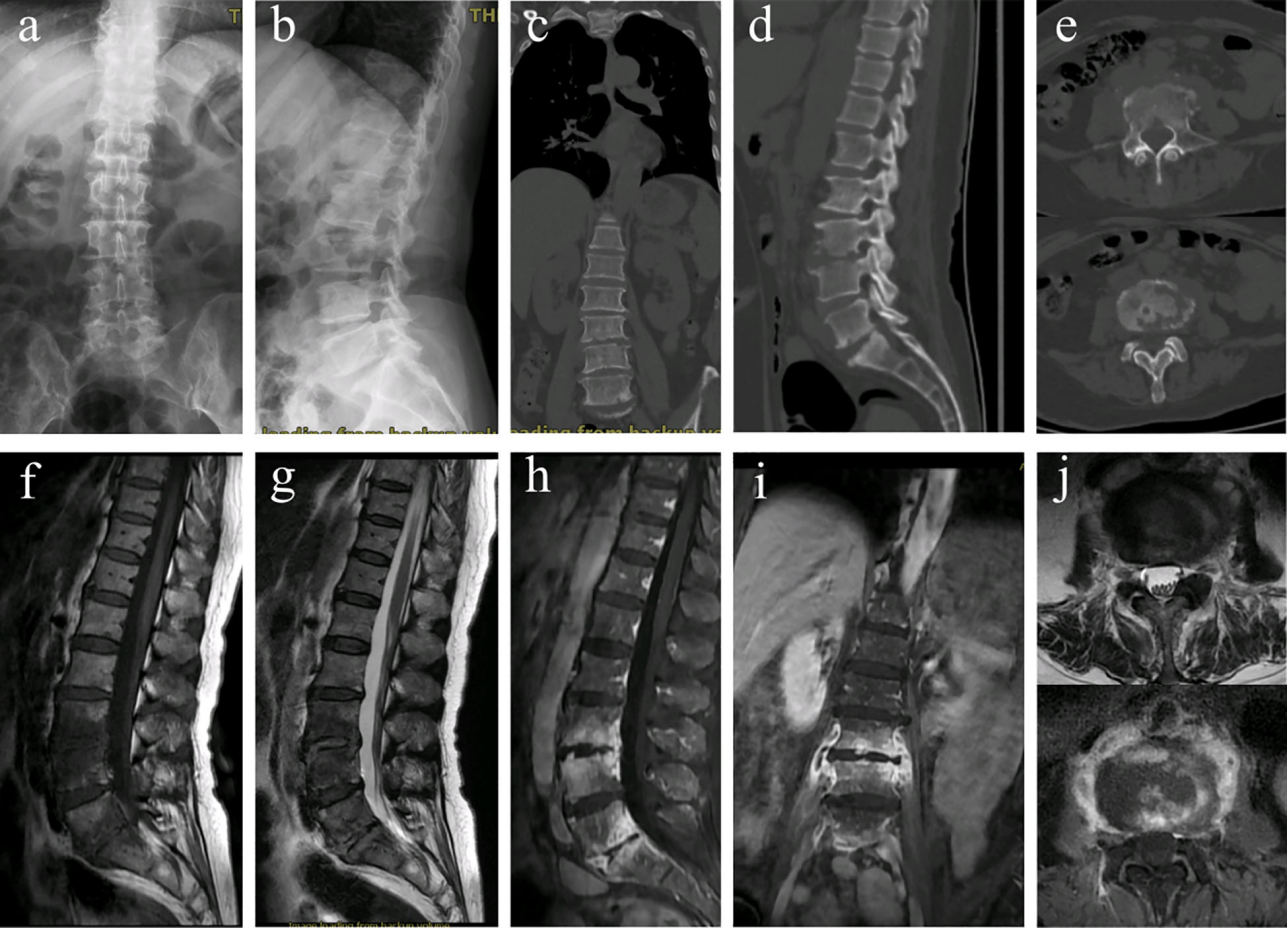
Representative pre-procedural imaging of a patient with lumbar brucellar spondylodiscitis (BS). **(a, b)** Preoperative anteroposterior and lateral lumbar radiographs. **(c–e)** Preoperative computed tomography (CT) scans showing osseous destruction of the opposing vertebral endplates at the L3–4 and L5–S1 levels. **(f–j)** Preoperative T2-weighted magnetic resonance imaging (MRI) demonstrating abnormally increased signal intensity within the intervertebral discs at L3–4 and L5–S1, accompanied by bone marrow edema in the adjacent vertebral bodies, which are typical imaging manifestations of BS.

**Figure 4:**
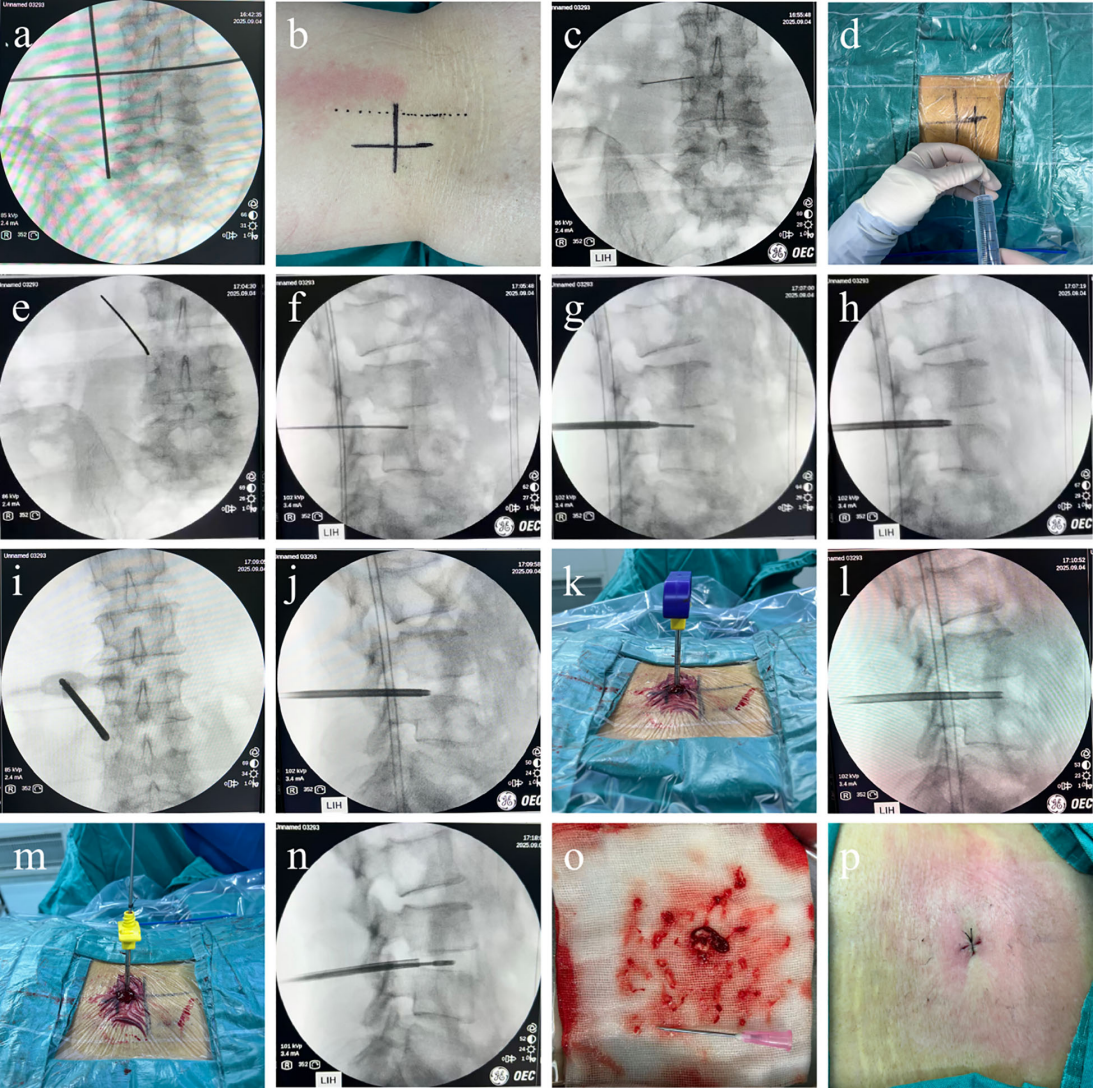
Presents the full procedural imaging sequence of percutaneous biopsy in a patient with lumbar brucellar spondylodiscitis. **(a, b)** show pre-procedural puncture site localization (marked as point P) under C-arm fluoroscopy guidance, while **(c, d)** demonstrate the local anesthesia procedure prior to the puncture. **(e)** displays the procedural localization point (marked as point S) for guidewire placement. After guidewire insertion via the extraforaminal approach under fluoroscopic guidance **(f)**, the hollow puncture cannula was sequentially advanced over the guidewire **(g, h)**, followed by insertion of the percutaneous aspiration biopsy needle through the cannula to obtain lesion tissue samples **(i, j, k, l)**, and then biopsy forceps were introduced to retrieve pathological tissue specimens **(m, n)**. **(o)** shows the intervertebral disc tissue specimen obtained from the lesion, and panel **(p)** presents the appearance of the puncture site immediately after the procedure.

The matched hollow puncture biopsy needle was coaxially inserted into the target area along the guide needle. After biplane fluoroscopy confirmed that the position was safe and accurate, the guide needle was withdrawn and the needle shaft was fixed, completing the puncture localization. **Figure 3** shows the preoperative imaging data of a patient with L3–4 BS, and **Figure 4** shows the full-procedure fluoroscopic images of the puncture for this patient.

#### 2.4.3 Specimen collection and peri-procedural safety management

After the needle tip was advanced into the target lesion, the hub of the hollow puncture needle was connected to a 20 mL syringe for pus aspiration, with the negative pressure controlled at ≤2 mL. For patients with no pus yield, a 2 mL negative pressure was maintained, and the needle was briefly advanced and retracted 2 to 3 times within the lesion to collect specimens; alternatively, a fine biopsy forceps could be inserted through the needle tract to harvest lesion tissue. All specimens were submitted for routine bacterial culture, Xpert assay, histopathological examination, and targeted next-generation sequencing (tNGS) detection.

Communication with the patient was maintained throughout the entire procedure. If symptoms of nerve root irritation occurred, the needle was withdrawn by 2 to 3 mm and re-advanced after angle adjustment. In the event of lower limb numbness or decreased muscle strength, the procedure was terminated immediately, the needle was fully withdrawn, and emergency magnetic resonance imaging (MRI) was performed to rule out nerve injury or hematoma. Neurological physical examinations were performed immediately after the procedure and at 24 hours post-procedure, and all procedure-related complications were documented.

#### 2.4.4 Individualized adjustment of puncture angle for different segments

The AP medial inclination angle is defined as the angle between the long axis of the puncture needle and the midline of the lumbar spinous processes, with medial tilt toward the midline designated as positive. The lateral craniocaudal inclination angle is defined as the angle between the long axis of the puncture needle and the horizontal line of the endplates of the target intervertebral space, with cephalad tilt designated as positive and caudad tilt as negative. Precautions for puncture at each segment are detailed in **Table 1**.

**Table 1:** Puncture angle adjustment and precautions for different lumbar segments.

Target segment	Anteroposterior (AP) Medial inclination angle	Lateral craniocaudal inclination angle
L1-2	10°–12°	0°– -5° (caudad tilt)
L2-3	12°–15°	0°
L3-4	15°–18°	0°
L4-5	20°–25°	0°
L5-S1	25°–30°	+10°– +15° (cephalad tilt)

### 2.5 Laboratory detection methods

#### 2.5.1 Histopathological examination

Puncture specimens were fixed in 10% neutral buffered formalin for 24 hours, embedded in paraffin, and serially sectioned at 4 μm for routine hematoxylin and eosin (HE) staining. Pathological abnormalities were classified into three categories: (1) tuberculous granuloma with or without caseous necrosis; (2) non-tuberculous granuloma; (3) acute or chronic inflammatory cell infiltration.

#### 2.5.2 Routine bacterial and anaerobic culture

Routine bacterial and anaerobic culture was performed in accordance with the standard operating procedures of the clinical microbiology laboratory. Specimens were inoculated onto blood agar, chocolate agar, and anaerobic blood agar plates. Aerobic plates were incubated at 35 °C in a 5% CO_2_ atmosphere, and anaerobic plates were incubated at 35 °C in an anaerobic environment, with the incubation period extended to 14 days. Positive strains were subjected to Gram staining, biochemical identification, and antimicrobial susceptibility testing.

#### 2.5.3 Xpert MTB/RIF assay

A 0.5 mL volume of puncture fluid or homogenized tissue specimen was collected. Sample processing buffer was added, and the mixture was incubated at room temperature for 15 minutes. The mixture was then transferred into an Xpert cartridge, which was loaded into the GeneXpert Dx System for fully automated nucleic acid extraction, amplification, and detection.

#### 2.5.4 Targeted next-generation sequencing

The tNGS assay was performed by Jinyu Medical Laboratory (Guangzhou, China) using the MetaPath Pathogen Capture Metagenomic Assay Kit (KingCreate, China). Specimens from all 3 participating centers were sent to this centralized laboratory within 24 hours of collection. The core technology utilizes species-specific biotinylated probes for hybridization capture (a targeted enrichment strategy distinct from broad-range 16S/ITS amplicon sequencing). The capture panel is built upon a comprehensive microbial database comprising 13, 214 bacteria, 9, 811 viruses, 3, 180 fungi, and 405 parasites sourced from NCBI. For clinical reporting in this study, interpretation was focused on pathogens associated with spinal infection.

The tNGS workflow comprised 4 main steps: (1) nucleic acid extraction from clinical specimens using the MetaPure DNA Extraction Kit; (2) library construction through enzymatic fragmentation, end-repair, adapter ligation, and PCR amplification, followed by hybridization capture with species-specific biotinylated probes for 2 hours; (3) high-throughput sequencing on the Illumina MiniSeq platform with single-end 100-bp reads, at an average depth of 1.0 million reads per sample; and (4) bioinformatics analysis, including quality control (bcl2fastq v2.20.0.422 and Fastp v0.23.1), host read depletion (alignment to hg38 using BWA v0.7.17), alignment to the microbial classification database, and RPM normalization.

Skin commensal bacteria and common environmental contaminants were excluded during the bioinformatics analysis phase through a curated exclusion list based on established clinical microbiology guidelines.

The positive detection thresholds were established based on the testing laboratory’s internally validated standards, which are concordant with the core principle of published clinical expert consensus that mycobacteria require more sensitive detection parameters than conventional bacteria due to their lower nucleic acid recovery efficiency. For bacteria, a threshold of ≥30 reads was applied. For mycobacteria, a lower threshold of ≥10 reads was used, reflecting the well-documented difficulty in DNA extraction from mycobacterial cell walls due to their high mycolic acid content. For fungi, viruses, and parasites, a threshold of ≥5 reads was applied, reflecting their smaller genome sizes and lower expected biomass in clinical specimens.

In addition to the pathogen-specific read-count thresholds, each batch incorporated a comprehensive quality-control system. Sequencing runs were required to yield a minimum of 300, 000 raw reads per specimen, with quality thresholds of Q20 ≥90% and Q30 ≥85%. A human reference gene (GAPDH) served as an internal control to verify adequate nucleic acid recovery and library quality; specimens were reprocessed if the GAPDH metrics (standardized read count and coverage) failed to meet the laboratory’s validated criteria. Probe capture efficiency, calculated as the ratio of probe-targeted microbial reads to total pathogen-assigned reads, was monitored with a batch-level threshold of 0.5. For batch acceptance, positive spike-in controls (bacterial, yeast, and viral phage) and negative extraction controls were included. The run was accepted only if at least two of the three positive controls met the species-specific criteria (RPM ≥10 or capture efficiency ≥50%) and the negative control showed no anomalous pathogen signal above the background threshold. As an additional safeguard, if mycobacterial sequences were detected in the negative control, low-read mycobacterial results from that batch were invalidated to exclude batch contamination. For clinical interpretation, pathogens were distinguished from background flora through the established bioinformatics pipeline that applied the aforementioned exclusion criteria, evaluated relative abundance (RPM), and required the organism’s read count to exceed the concurrent negative control by a prespecified fold-change threshold.

In routine clinical practice, tNGS was performed concurrently with histopathology, routine bacterial culture, and Xpert MTB/RIF assay, and results were subsequently returned to the clinical team to inform antimicrobial therapy selection. For the purpose of this retrospective study, tNGS results were extracted from the archived clinical laboratory reports in the electronic medical record system after the final diagnosis had been independently established, to avoid incorporation bias in the diagnostic yield analysis. The reports provided clinically interpreted pathogen identification based on the aforementioned thresholds and exclusion criteria.

### 2.6 Reference standard for final clinical diagnosis

The final diagnosis was independently established by at least two senior spine surgeons with more than 10 years of clinical experience, based on clinical manifestations, imaging findings, histopathology, routine bacterial culture, and serological results. tNGS results were explicitly excluded from the determination of the final diagnosis to avoid incorporation bias. This reference standard was established as a study-specific diagnostic criterion independent of routine clinical decision-making. Discrepancies were resolved through arbitration by a third chief physician. For the research analysis, the tNGS data were compared against this locked reference standard. A definitive diagnosis of STB or BS was established if the patient met either one of the etiological confirmation criteria or all of the corresponding clinical diagnostic criteria, with other spinal diseases excluded. A definitive diagnosis of PS required fulfillment of the corresponding diagnostic criteria, with tuberculosis and Brucella infection ruled out. The specific criteria are detailed below:

#### 2.6.1 STB:etiological confirmation criteria

① Typical tuberculous granuloma with caseous necrosis and positive acid-fast staining observed on histopathological examination of puncture specimens; ② Positive Xpert MTB/RIF assay of the specimen; ③ Isolation of Mycobacterium tuberculosis complex from specimen culture. Clinical Diagnostic Criteria: Clinical and imaging manifestations consistent with tuberculosis, positive T-SPOT.TB result, elevated inflammatory biomarkers, and exclusion of other spinal infections and tumors (Fuentes Ferrer et al., 2012).

#### 2.6.2 BS:etiological confirmation criteria

① Isolation of Brucella species from specimen culture; ② Standard tube agglutination test (SAT) titer ≥1:100, or a 4-fold or greater increase in titer during the disease course. Clinical Diagnostic Criteria: Documented epidemiological exposure history, clinical and imaging manifestations consistent with BS, elevated inflammatory biomarkers, and exclusion of other spinal infections and tumors (Abulizi et al., 2021).

#### 2.6.3 PS:etiological confirmation criterion

Definitive pyogenic pathogenic bacteria isolated from specimen culture. Clinical Diagnostic Criteria: Acute onset with typical infectious manifestations such as low back pain and fever, imaging manifestations consistent with PS, significantly elevated inflammatory biomarkers, and exclusion of tuberculosis, Brucella infection, and non-infectious spinal diseases (Cheung and Luk, 2012).

### 2.7 Statistical analysis

#### 2.7.1 Sample size consideration

Given the retrospective consecutive enrollment design, the sample size was determined by the number of eligible patients treated at the three centers during the study period (January 2024 to January 2026). Nevertheless, a sample size justification was performed using the standard formula for estimating a single proportion in diagnostic accuracy studies (n = Z_1-_α/_2_² × *P* × (1−*P*)/W²). Assuming an expected sensitivity of 85% for the index test, a permissible margin of error (W) of 10%, and a two-sided confidence level of 95%, the minimum required sample size was calculated to be 49 patients. The enrolled 94 patients exceeded this requirement.

All statistical analyses and graphical visualizations in this study were performed using R software version 4.5.1. Measurement data were tested for normality using the Shapiro-Wilk test. Normally distributed data were presented as mean ± standard deviation (SD), and one-way analysis of variance (ANOVA) was used for comparisons among multiple groups. Non-normally distributed data were presented as median (interquartile range, IQR) *[M (Q1, Q3)]*, and the Kruskal-Wallis H test was applied for multi-group comparisons. Categorical data were expressed as frequency (percentage) [n (%)], and inter-group comparisons were performed using the chi-square (*χ²*) test, with Fisher’s exact test used when the expected frequency was less than 5.

To ensure methodological validity, the reference standard (final comprehensive clinical diagnosis) was established independently without incorporation of tNGS results, thereby enabling unbiased estimation of diagnostic performance metrics (detection yield).With the final comprehensive clinical diagnosis as the reference standard, the positive rate and sensitivity (with exact 95% CIs) of each etiological detection method were calculated using 2×2 contingency tables. For paired comparisons of etiological methods from the same subject, McNemar’s exact test was used; the overall comparison across the three etiological methods was performed using Cochran’s Q test. The agreement between tNGS and traditional methods was evaluated using Cohen’s kappa coefficient, with the strength of agreement graded in accordance with universally accepted standard criteria. All statistical tests were two-sided, and a *P*-value < 0.05 was considered statistically significant.

## 3 Results

### 3.1 Preoperative baseline data and clinical characteristics of patients

A total of 94 patients with SI who met the pre-specified inclusion and exclusion criteria were enrolled in this study, including 43 cases (45.74%) from the leading center, 30 cases (31.91%) and 21 cases (22.34%) from the other two participating centers, respectively. The overall age of the patients was 60.80 ± 10.32 years (range 28 to 82 years), with 57 males (60.64%) and 37 females (39.36%). The final clinical diagnoses were STB in 32 cases (34.04%), BS in 33 cases (35.11%), and PS in 29 cases (30.85%).Baseline demographic and clinical characteristics across the three centers were broadly comparable, with no significant differences in age, underlying comorbidities, epidemiological exposure history, or etiological composition (all *P* > 0.05). A statistically significant difference in gender distribution was observed (*P* = 0.0010), reflecting heterogeneous patient sources across the three centers; this was not considered a confounding factor for the diagnostic accuracy of the index test. Preoperative laboratory and imaging findings were also comparable among the three centers (all *P* > 0.05), supporting the validity of pooling multicenter data; detailed baseline data are presented in **Table 2**, and detailed laboratory and imaging results are provided in **Supplementary Tables S1**, **S2**.

**Table 2:** Comparison of baseline clinical characteristics of patients across the 3 participating centers.

Index	Center A (n=43)	Center B (n=30)	Center C (n=21)	*P*-value
Age (years)	60.21 ± 11.04	61.4 ± 10.56	61.55 ± 8.77	0.8444
Sex				
- Male, n (%)	31 (72.09)	10 (33.33)	16 (76.19)	0.0010
- Female, n (%)	12 (27.91)	20 (66.67)	5 (23.81)	
Hypertension, n (%)	8 (18.60)	6 (20.00)	6 (28.57)	0.6441
Diabetes mellitus, n (%)	4 (9.30)	3 (10.00)	2 (9.52)	0.9950
Animal exposure history, n (%)(raw beef and mutton consumption, unprotected livestock parturition handling, residence in pastoral areas, etc.)	6 (13.95)	4 (13.33)	3 (14.29)	0.9948
Previous history of tuberculosis, n (%)	5 (11.63)	3 (10.00)	3 (14.29)	0.8958
Visual Analogue Scale (VAS) score for low back pain, points	6.33 ± 1.41	5.99 ± 2.51	6.03 ± 2.03	0.7260
Fever (body temperature ≥37.5°C), n (%)	29 (67.44)	23 (76.67)	16 (76.19)	0.6213
Final clinical diagnosis				
- STB	15 (34.88)	11 (36.67)	6 (28.57)	0.5695
- BS	18 (41.86)	8 (26.67)	7 (33.33)	
- PS	10 (23.26)	11 (36.67)	8 (38.10)	

### 3.2 Puncture procedure indicators and safety analysis

**Figure 5:**
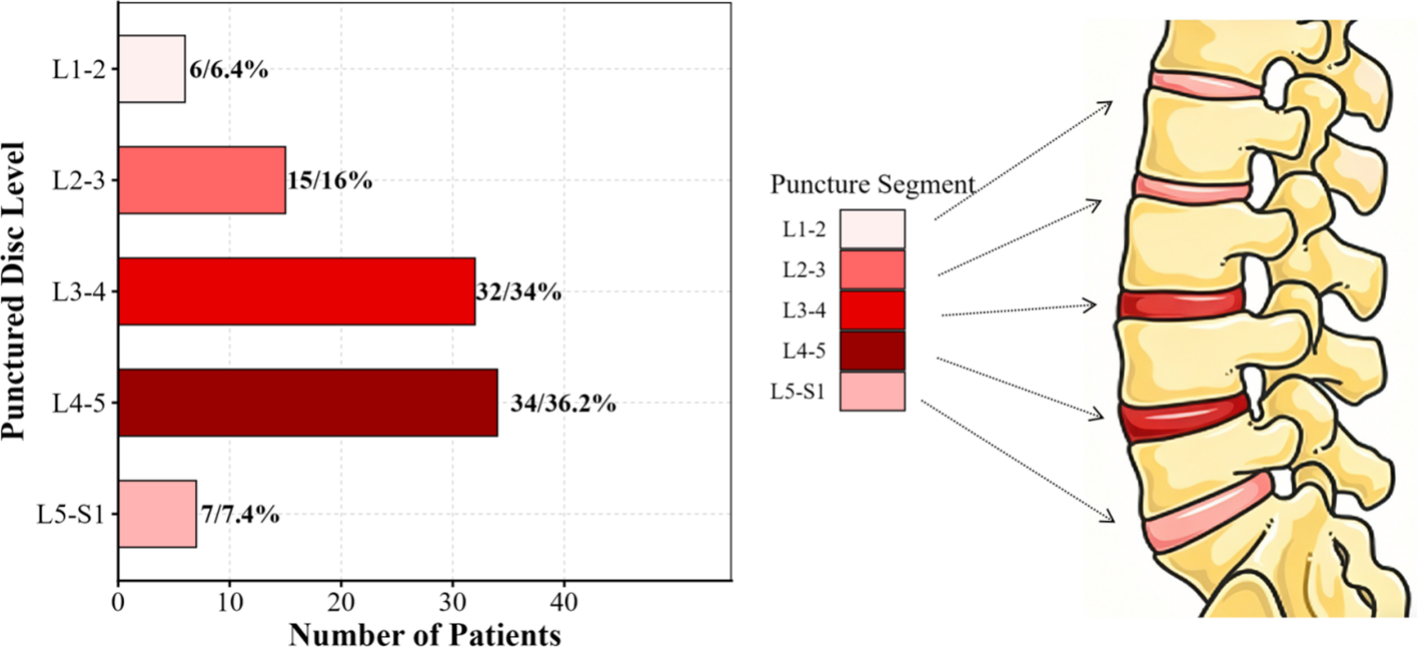
Heat map showing the distribution of punctured lesional intervertebral disc segments in the entire patient cohort.

All 94 patients enrolled in this study successfully underwent puncture biopsy and obtained qualified specimens. The distribution of punctured lesion disc segments across the 3 centers is shown in **Figure 5**. A comparison of puncture procedure–related indicators among the 3 centers is detailed in **Table 3**. Between-group comparisons demonstrated that the difference in total procedure time among the 3 centers approached statistical significance (*P* = 0.0504), the difference in mean fluoroscopy times was statistically significant (*P* = 0.0002), with the leading center (Center A) requiring the fewest mean exposures (13.95 ± 1.56), followed by Center B (15.07 ± 2.48) and Center C (16.14 ± 1.71), suggesting a potential learning curve effect. The difference in intraoperative blood loss was not statistically significant (*P* = 0.0695).

**Table 3:** Comparison of puncture procedure indicators and complications across 3 centers.

Index	Center A (n=43)	Center B (n=30)	Center C (n=21)	*P*-value
Total procedure time, min	16.23 ± 3.85	17.76 ± 3.24	18.21 ± 2.46	0.0504
Mean fluoroscopy times, n	13.95 ± 1.56	15.07 ± 2.48	16.14 ± 1.71	0.0002
Intraoperative blood loss, mL	11.50 ± 4.29	12.20 ± 4.31	14.38 ± 5.69	0.0695
Puncture-related complications, n (%)	3 (6.97)	2 (6.67)	2 (9.52)	-
Immediate complications, n (%)	3 (6.97)	2 (6.67)	2 (9.52)	-
- Minor oozing at puncture site, n (%)	2 (4.65)	1 (3.33)	1 (4.76)	-
- Intraoperative nerve irritation, n (%)	1 (2.32)	1 (3.33)	1 (4.76)	-
- Dural rupture/CSF leakage, n (%)	0	0	0	-
- Major vascular injury, n (%)	0	0	0	-
Delayed complications (24h post-op), n (%)	0	0	0	-
- Severe neurological sequelae, n (%)	0	0	0	-
- Puncture site infection, n (%)	0	0	0	-

The overall rate of puncture-related complications in the entire cohort was 7.45%, all of which were mild adverse events occurring intraoperatively or immediately postoperatively. These included mild bleeding at the puncture site in 4 cases and transient nerve root irritation during the procedure in 3 cases; all these mild complications resolved rapidly after symptomatic treatment. No severe adverse events, such as dural tear/cerebrospinal fluid (CSF) leakage, major vascular injury, permanent neurological dysfunction, or puncture site infection, occurred in the entire cohort. These findings suggest that the puncture approach was feasible and appeared safe in this retrospective cohort, though the single-operator-per-center design and absence of a prospective control group preclude definitive conclusions regarding broad clinical safety.

### 3.3 Overall distribution of histopathological and etiological test results

**Figure 6:**
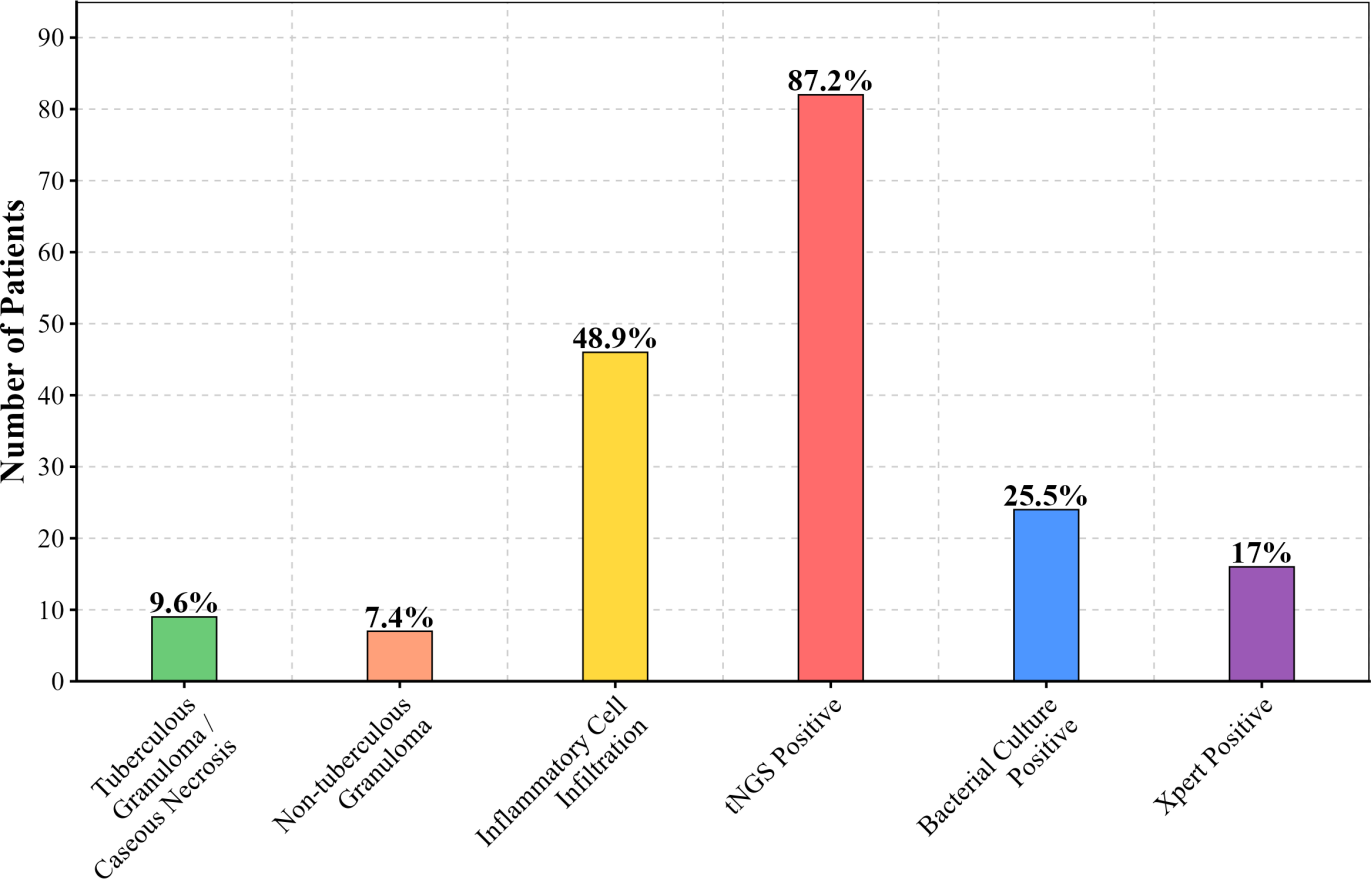
Bar chart illustrating the distribution of positive rates for pathological and etiological examinations in the entire patient cohort.

The comparison of overall histopathological and etiological test results for patients in the entire cohort across the 3 participating centers is presented in **Figure 6**. There were no statistically significant differences in the distribution of histopathological findings and etiological test results among the three groups (all *P* > 0.05), with detailed data shown in **Table 4**.

**Table 4:** Overall distribution of histopathological and etiological findings.

Category	Index	Center A (n=43)	Center B (n=30)	Center C (n=21)	*P*-value	Total (n=94)
Histopathology	Tuberculous granuloma/caseous necrosis	4 (9.30)	3 (10.00)	2 (9.52)	0.9950	9 (9.57)
	Other granulomas	3 (6.98)	2 (6.67)	2 (9.52)	0.9177	7 (7.45)
	Inflammatory cell infiltration	19 (44.19)	16 (53.33)	11 (52.38)	0.6976	46 (48.94)
Etiology	tNGS positive	38 (88.37)	26 (86.67)	18 (85.71)	0.9502	82 (87.23)
	- *Mycobacterium tuberculosis*	14 (32.56)	8 (26.67)	5 (23.81)	0.7340	27 (28.72)
	- *Brucella* species	16 (37.21)	7 (23.33)	6 (28.57)	0.4359	29 (30.85)
	- *Staphylococcus aureus*	5 (11.63)	6 (20.00)	4 (19.05)	0.5723	15 (15.96)
	- *Escherichia coli*	2 (4.65)	2 (6.67)	1 (4.76)	0.9235	5 (5.32)
	- *Streptococcus pneumoniae*	1 (2.33)	1 (3.33)	1 (4.76)	0.3977	3 (3.19)
	- *Streptococcus haemolyticus*	0 (0.00)	1 (3.33)	1 (4.76)	0.3977	2 (2.13)
	- *Klebsiella pneumoniae*	1 (2.33)	0 (0.00)	0 (0.00)	0.5491	1 (1.06)
	routine bacterial culture positive	11 (25.58)	7 (23.33)	6 (28.57)	0.9147	24 (25.53)
	- *Brucella* species	7 (16.28)	3 (10.00)	3 (14.29)	0.7448	13 (13.83)
	- *Staphylococcus aureus*	1 (2.33)	2 (6.67)	2 (9.52)	0.4470	5 (5.32)
	- *Escherichia coli*	1 (2.33)	1 (3.33)	1 (4.76)	0.8720	3 (3.19)
	- *Streptococcus pneumoniae*	1 (2.33)	1 (3.33)	0 (0.00)	0.7139	2 (2.13)
	- *Klebsiella pneumoniae*	1 (2.33)	0 (0.00)	0 (0.00)	0.5491	1 (1.06)
	Xpert MTB/RIF positive	8 (18.60)	6 (20.00)	2 (9.52)	0.5768	16 (17.02)
	- *Mycobacterium tuberculosis*	8 (18.60)	6 (20.00)	2 (9.52)	0.5768	16 (17.02)

The category of Inflammatory cell infiltration, which represents Infection-indicative histopathology, includes two specific pathological changes: Tuberculous granuloma/caseous necrosis and Non-tuberculous granuloma. Other pathological findings without evidence of inflammatory cell changes were not included in this table.

For histopathological examination, 48.94% (46/94) of cases showed non-specific inflammatory cell infiltration, and only 17.02% of cases obtained a specific etiological diagnosis via histopathology (9 cases with tuberculous granuloma with caseous necrosis, 7 cases with non-tuberculous granuloma).

**Figure 7:**
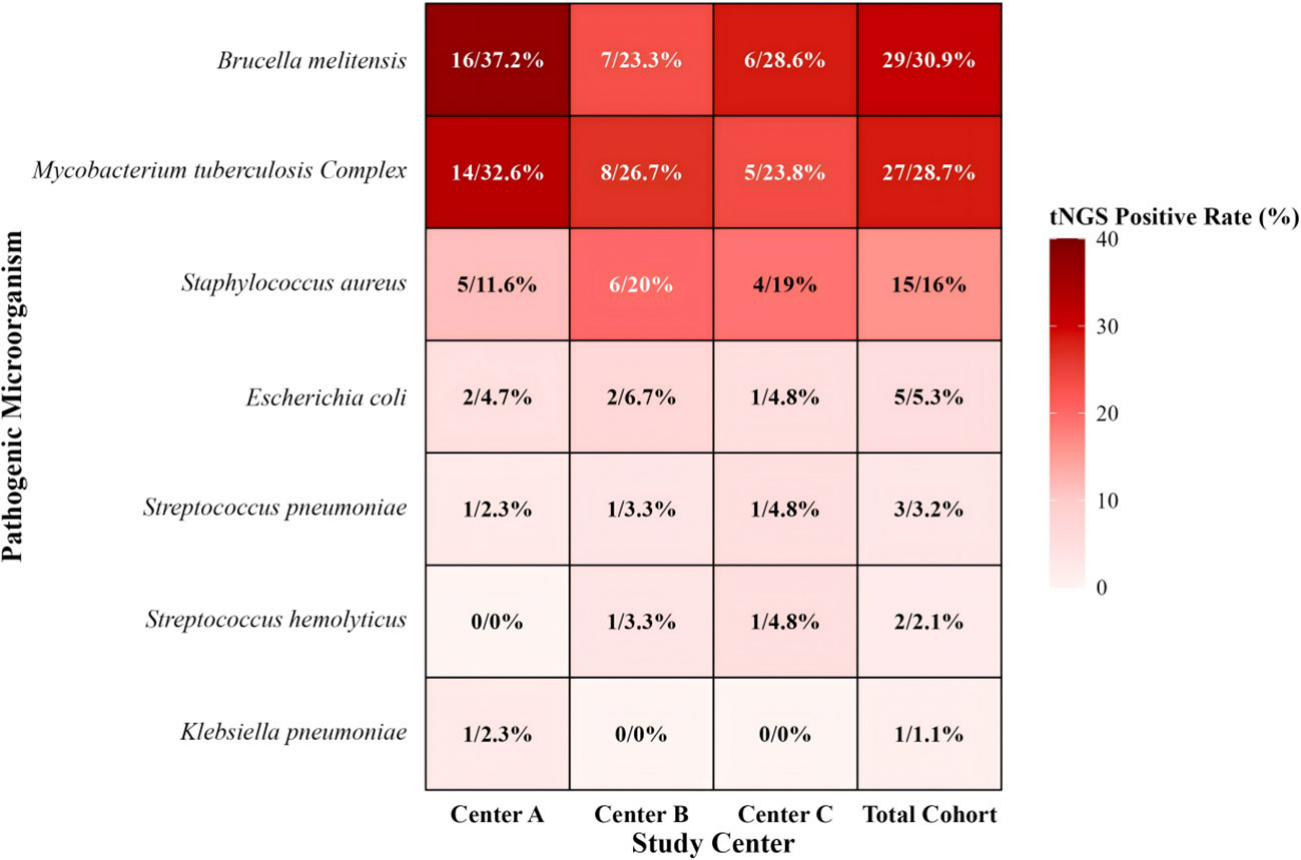
Heatmap of identified pathogenic species and their distribution by tNGS in the entire patient cohort.

For etiological detection, the overall positive rate of tNGS was 87.23% (82/94), the highest among all detection methods. The detected pathogenic spectrum was dominated by Mycobacterium tuberculosis (27 cases, 28.72%), Brucella species (29 cases, 30.85%), and Staphylococcus aureus (15 cases, 15.96%), which was highly consistent with the etiological distribution of the final clinical diagnoses. The detailed species and distribution of detected pathogens are shown in **Figure 7**. The overall positive rate of routine bacterial culture was 25.53% (24/94), which was significantly lower than that of tNGS (*P* < 0.0001), with Brucella species having the highest detection rate (13 cases, 13.83%). The overall positive rate of the Xpert MTB/RIF assay was 17.02% (16/94), which was only detected in STB cases, with a positive rate of 50.00% among the 32 patients with STB.

### 3.4 Comparison of diagnostic performance between histopathological and etiological testing

Using the final comprehensive clinical diagnosis as the reference standard, the etiological detection yield of each method is presented in **Table 5**. The detection yield of tNGS was 87.23% (95% CI: 79.0%--92.5%), significantly higher than that of routine bacterial culture (25.53%), Xpert MTB/RIF assay (17.02%), and specific histopathological diagnosis (17.02%) (all *P* < 0.0001, McNemar’s test). The overall detection yield of any etiological test was 91.49% (95% CI: 84.5%--96.0%). The paired 2×2 comparisons against histopathological inflammatory cell infiltration are detailed in **Table 6**. Among the 46 patients with histopathology showing only non-specific inflammatory cell infiltration, the etiology confirmation rate of combined detection was 95.65% (**Table 7**).

**Table 5:** Etiological detection yield of each method relative to the final clinical diagnosis (n=94).

Detection method	Positive n (%)	Detection yield* % (95% CI)
Histopathology — inflammatory cell infiltration	46 (48.94)	48.94 (39.1–58.9)
Histopathology — specific diagnosis†	16 (17.02)	17.02 (10.8–25.9)
tNGS	82 (87.23)	87.23 (79.0–92.5)
Routine bacterial culture	24 (25.53)	25.53 (17.8–35.2)
Xpert MTB/RIF assay	16 (17.02)	17.02 (10.8–25.9)
Any etiological test positive	86 (91.49)	91.49 (84.5–96.0)

Detection yield (sensitivity) was calculated using the final comprehensive clinical diagnosis as the reference standard. Because all enrolled patients had confirmed infection, specificity, PPV, and NPV are not applicable for overall infection diagnosis; see **Table 8** for subtype-specific metrics.

†Specific histopathology includes tuberculous granuloma with caseous necrosis (n=9) and non-tuberculous granuloma (n=7).

**Table 6:** Paired 2×2 contingency tables for etiological detection methods against histopathological inflammatory cell infiltration.

Comparison	HP(+)/Test(+)	HP(+)/Test(−)	HP(−)/Test(+)	HP(−)/Test(−)	McNemar *P*-value
tNGS vs. HP	42	4	40	8	<0.0001
Routine bacterial culture vs. HP	9	37	15	33	0.0021
Xpert MTB/RIF vs. HP	10	36	6	42	<0.0001

HP, histopathological inflammatory cell infiltration. The denominator for HP(+) is n=46; for HP(−) is n=48. McNemar’s test was used for paired comparisons. These 2×2 tables are presented for method comparison only; HP is not used as a diagnostic gold standard.

**Table 7:** Supplementary diagnostic value of etiological testing in cases with pathologically indicated “inflammatory cell infiltration”.

Final clinical diagnosis	No. of cases	Definitive etiology by tNGS	Definitive etiology by routine bacterial culture	Xpert positive (STB only)	Definitive etiology by any etiological test
STB	17	14 (82.35%)	0 (0.00%)	10 (58.82%)	16 (94.12%)
BS	15	15 (100.00%)	3 (20.00%)	—	15 (100.00%)
PS	14	13 (92.86%)	6 (42.86%)	—	13 (92.86%)
Total	46	42 (91.30%)	9 (19.57%)	10 (21.74%)	44 (95.65%)

Notably, among the 54 patients (57.45%) in whom both routine bacterial culture and Xpert MTB/RIF yielded negative results, tNGS provided the sole positive etiological diagnosis in 46 patients (48.94%) and had informed the selection of targeted anti-infective therapy during routine clinical care. The distribution by pathogen type was 13/32 STB (40.62%), 18/33 BS (54.55%), and 15/29 PS (51.72%). For the remaining 40 patients (42.55%) in whom at least one conventional method was also positive, tNGS served to confirm the etiology or identify co-pathogens in 36 patients (38.30%), while 4 patients (4.26%) had negative tNGS results. Collectively, tNGS results had contributed to the anti-infective treatment decision-making process in 86 patients (91.49%) during routine clinical care, while the remaining 8 patients (8.51%) with all-negative etiological assays were managed based on clinical judgment and follow-up.

### 3.5 Stratified analysis of detection rates of each test method across different etiological types

The subtype-specific diagnostic accuracy of each test method, stratified by the final pathogen type (STB, BS, and PS), is presented in **Table 8**. tNGS maintained high sensitivity across all three major subtypes (STB: 84.38%, BS: 87.88%, PS: 89.66%) with 100% specificity in each subtype, demonstrating its broad-spectrum etiological diagnostic capability without cross-reactivity to alternative pathogens.

**Table 8:** Detection rates of different etiological assays in various types of spinal infection.

Assay method	Tuberculous (STB, n=32)	Brucellar (BS, n=33)	Pyogenic (PS, n=29)
	Se/Sp/PPV/NPV (%)	Se/Sp/PPV/NPV (%)	Se/Sp/PPV/NPV (%)
tNGS	84.38/100/100/92.54	87.88/100/100/93.85	89.66/100/100/95.59
Routine bacterial culture	0.00/100/—/65.96	39.39/100/100/75.31	37.93/100/100/78.31
Xpert MTB/RIF	50.00/100/100/79.49	0.00/100/—/64.89	0.00/100/—/69.15
Any positive etiological test	90.62/100/100/95.38	93.94/100/100/96.83	89.66/100/100/95.59
Histopathology (tuberculous granuloma/caseous necrosis)	28.13/100/100/72.94	—/—/—/—	—/—/—/—
Histopathology (non-tuberculous granuloma)	—/—/—/—	21.21/100/100/70.11	—/—/—/—

Se, sensitivity; Sp, specificity; PPV, positive predictive value; NPV, negative predictive value. Specificity was calculated using patients with alternative final diagnoses as the non-diseased control group. Specificity, PPV, and NPV were calculated for subtype differentiation (i.e., discrimination among STB, BS, and PS) using patients with alternative final diagnoses as the non-diseased control group. These metrics do not apply to the diagnosis of spinal infection overall, as all enrolled patients had confirmed infection.

Traditional etiological detection methods showed marked pathogen-dependent performance: the sensitivity of routine bacterial culture was 39.39% and 37.93% in BS and PS, respectively, but 0% in STB; the Xpert MTB/RIF assay achieved 50.00% sensitivity in STB but was not applicable to BS or PS; specific histopathological findings (tuberculous granuloma with caseous necrosis and non-tuberculous granuloma) yielded sensitivities of 28.13% and 21.21% in STB and BS, respectively, with no diagnostic performance in PS. Notably, all methods except nonspecific inflammatory cell infiltration achieved 100% specificity, indicating that positive results from routine bacterial culture, Xpert, and specific granulomatous pathology are highly reliable when obtained, albeit with limited sensitivity.

### 3.6 Supplementary diagnostic value of etiological testing in cases with pathological evidence of “inflammatory cell infiltration”

Among the 46 cases where histopathology only indicated non-specific inflammatory cell infiltration and failed to confirm the etiology, the final clinical diagnoses were STB in 17 cases, BS in 15 cases, and PS in 14 cases, which was consistent with the overall etiological distribution of the entire cohort. This finding indicated that non-specific inflammatory cell infiltration is a common pathological manifestation of all subtypes of spinal infection.

In this subgroup of cases, etiological testing exhibited an extremely high supplementary diagnostic value, with detailed data presented in **Table 7**. tNGS confirmed the etiology in 42 cases (91.30%), with a 100% diagnostic rate in BS cases; routine bacterial culture confirmed the etiology in 9 cases (19.57%); the Xpert MTB/RIF assay achieved a positive rate of 58.8% in STB cases. Combined detection using any of the etiological methods confirmed the etiology in 44 cases (95.65%), and only 2 cases (4.35%) were retrospectively diagnosed by the final comprehensive clinical diagnosis, including 1 case in the STB subgroup and 1 case in the PS subgroup.

### 3.7 Agreement analysis between different etiological detection methods

Cohen’s kappa test was used to evaluate the agreement between tNGS and traditional etiological detection methods, with the results shown in **Table 9**. When calculated using the entire cohort of 94 patients as the denominator, the concordance rates between tNGS and Xpert MTB/RIF assay (for tuberculosis detection), routine bacterial culture (for brucellosis detection), and routine bacterial culture (for pyogenic bacteria detection) were 84.04%, 78.72%, and 84.04%, respectively. The corresponding kappa coefficients were 0.556 (95% CI: 0.349–0.738), 0.411 (95% CI: 0.209–0.603), and 0.515 (95% CI: 0.306–0.702), all indicating moderate agreement (all *P* < 0.001). Across the three comparisons, the number of positive cases detected by tNGS was higher than that detected by Xpert MTB/RIF for tuberculosis (27 vs. 16), and higher than that detected by routine bacterial culture for brucellosis (29 vs. 13) and pyogenic bacteria (26 vs. 11). Notably, the discordant cases across all three comparisons predominantly reflected tNGS-positive/traditional-method-negative patterns, indicating that the moderate agreement primarily reflects the incremental sensitivity of tNGS rather than bidirectional contradictory errors.

**Table 9:** Agreement analysis between different etiological methods.

Comparison group	Total cases	tNGS+ (n)	Reference+ (n)	Concordant+ (n)	Concordant− (n)	Concordant cases (n)	Concordance rate (%)	Kappa	95% CI	*P*-value
tNGS (TB) vs. Xpert MTB/RIF	94	27	16	14	65	79	84.04	0.556	0.349–0.738	<0.0001
tNGS (Brucella) vs. routine bacterial culture (Brucella)	94	29	13	11	63	74	78.72	0.411	0.209–0.603	0.0004
tNGS (Pyogenic) vs. routine bacterial culture (Pyogenic)	94	26	11	11	68	79	84.04	0.515	0.306–0.702	<0.0001

Cohen’s kappa coefficient was calculated using the entire cohort of 94 patients as the denominator for all comparisons. The observed agreement (Po) and expected agreement (Pe) were 84.04% and 64.03% for TB, 78.72% and 63.85% for Brucella, and 84.04% and 67.11% for Pyogenic, respectively. The 95% confidence intervals were derived using the bootstrap method (10, 000 iterations). Routine bacterial culture was not optimized for mycobacteria detection (e.g., Lowenstein-Jensen medium or BACTEC MGIT system were not routinely used). TB, tuberculosis; Xpert MTB/RIF, Xpert Mycobacterium tuberculosis/rifampicin resistance assay.

## 4 Discussion

Early SI lacks specific clinical manifestations and presents primarily with atypical symptoms including low back pain and low-grade fever. Conventional radiography and computed tomography (CT) rarely show characteristic changes within the first 4 weeks of the disease course, resulting in delayed diagnosis of more than 3 weeks in approximately 40% of affected patients. Timely early diagnosis and intervention can significantly reduce the incidence of devastating clinical outcomes, including permanent neurological deficit, spinal deformity, and sepsis (Niu et al., 2022; Gao et al., 2025). The 2024 Delphi consensus from the Spine Section of the European Association of Neurosurgical Societies (EANS) explicitly states that if blood culture results are negative and subsequent treatment is non-surgical or surgical intervention is delayed, prompt puncture biopsy should be performed to identify the pathogen and guide antimicrobial therapy (Kramer et al., 2025).

SI lesions frequently involve both the vertebral bone and intervertebral disc simultaneously. Clinical vertebral puncture biopsy is most commonly performed via the transpedicular bony channel to obtain bone tissue from the center of the vertebral body. However, restricted by the fixed trajectory of the bony channel, it is difficult to access lesions near the inferior edge of the vertebral body and the endplates. This inherent anatomical limitation in the scope of sampling and types of tissue obtained is one of the core reasons for the low positive rate of pathogen detection (Sertic et al., 2019; Zamarud et al., 2024). In a biopsy cohort study enrolling 310 patients with suspected vertebral osteomyelitis-discitis, Malik et al. (2025) reported that the overall positive rate of routine bacterial culture from percutaneous puncture biopsy in SI was 34%, with no significant impact of preoperative antibiotic exposure on the overall positive rate. Specifically, the positive rate was 36% for intervertebral disc sampling and 42% for lesion aspiration biopsy, which were significantly higher than 8% for simple vertebral bone biopsy and 29% for core needle bone biopsy. A study by Mazarakis et al. (2023) similarly confirmed that when biopsy specimens of SI contained intervertebral disc tissue, the positive rate of etiological diagnosis was significantly increased to 47%, compared with only 15% for simple vertebral bone sampling. Although transpedicular vertebral biopsy has a favorable safety profile, its excessively low pathogen detection efficiency renders targeted, accurate puncture biopsy of the intervertebral disc an essential component of etiological diagnosis for early SI. The aforementioned studies, as well as the percutaneous intervertebral disc puncture biopsy commonly used in clinical practice, mostly adopt the far posterolateral approach with an entry point 10–15 cm or more lateral to the midline of the spinous processes. The puncture and localization workflow of this approach is homologous to the preoperative targeted puncture technique for transforaminal endoscopy. However, this approach requires large-angle oblique needle insertion with a long puncture trajectory that traverses multiple layers of the paraspinal muscles, increasing the risk of soft tissue injury-related complications. Meanwhile, accurate target localization requires repeated fluoroscopic adjustment due to artifact from the muscular and soft tissue along the trajectory. Even when the puncture needle is successfully placed into the intervertebral disc, the operable adjustment range within the disc is still significantly limited, which easily leads to insufficient biopsy sample volume and poor lesion representativeness, ultimately compromising the accuracy of histopathological diagnosis and the positive rate of pathogen detection (Madhavan et al., 2017; Ang et al., 2019; Pan et al., 2020).

The puncture approach developed in this study leverages the anatomical safe window of the lateral lumbar intervertebral foramen, with the skin entry point positioned at the paraspinal aponeurosis transition zone 0.8–1.5 cm lateral to the spinous process midline, and a maximum medial inclination angle controlled within 30°. Its puncture trajectory is approximately 50% shorter than that of the conventional far posterolateral approach, and it does not require traversal of the psoas major muscle throughout the entire procedure, inherently reducing the risk of muscle injury and nerve root irritation based on its anatomical design. Meanwhile, it enables direct targeting of the intervertebral space and sub-endplate lesions, allowing accurate acquisition of core lesion tissue from early SI, and circumvents the inherent limitation of insufficient specimen quality associated with the transpedicular approach.

There were no statistically significant differences in total procedure time and intraoperative blood loss among the three participating centers (*P* = 0.0504 and *P* = 0.0695, respectively), with only a statistically significant between-group difference in the mean number of fluoroscopy exposures (*P* = 0.0002). This finding likely reflects a learning curve effect, as the leading center (Center A) required the fewest mean exposures (13.95 ± 1.56), followed by Center B (15.07 ± 2.48) and Center C (16.14 ± 1.71). This operator-dependent gradient reflects the learning curve inherent to the technique, and it should be candidly acknowledged that structured training is required before operators of varying experience can achieve consistent proficiency. The overall incidence of puncture-related complications in the entire cohort was only 7.45%, and all adverse events were mild, including minor oozing at the puncture site and transient nerve root irritation. No severe complications or delayed adverse events occurred in the cohort, supporting the feasibility and apparent safety of this procedure in this selected retrospective cohort, though prospective validation involving multiple operators per center is necessary before widespread clinical adoption.

For decades, histopathological examination has long been regarded as one of the gold standards for the diagnosis of SI, yet this traditional diagnostic system has substantial limitations in its clinical value for early-stage SI (Panico et al., 2023). The pathological changes of early SI are dominated by diffuse inflammatory cell infiltration, and rarely develop specific pathological features such as tuberculous granuloma with caseous necrosis and Brucella-associated epithelioid granuloma (Wu et al., 2023; Ma et al., 2026). Data from this study showed that only 17.02% of cases obtained a specific etiological diagnosis via histopathology, and nearly half (48.94%) of cases only indicated non-specific inflammatory cell infiltration, which failed to distinguish disease subtypes, let alone provide information such as pathogen species. Histopathological diagnosis can only achieve preliminary differentiation between infectious and non-infectious lesions, and cannot provide precise guidance for the formulation of clinical antimicrobial regimens (Li et al., 2020).

The limitations of routine bacterial culture are equally prominent, with an overall positive rate of only 25.53% in this study, which is consistent with the positive rate range of 20%–30% reported in international guidelines. Specifically, no mycobacterial growth was observed among the 32 patients with a confirmed diagnosis of STB. This is because the culture of Mycobacterium tuberculosis requires a specialized culture medium in a biosafety level 3 laboratory(BSL-3), requirements that cannot be met by the routine bacterial culture available in general hospitals. The positive rate of culture was only 39.4% among the 33 patients with BS. Even though the incubation period was extended to 14 days in this study to cover slow-growing pathogens, it was still difficult to break through the bottleneck in positive rate, which completely failed to meet the timeliness requirements for the early diagnosis of SI. T-SPOT.TB only achieved a positive rate of 65.63% in patients with a final confirmed diagnosis of STB, with a false-negative rate of 34.37%. The Brucella SAT only had a positive rate of 51.52% in patients with confirmed BS, with a false-negative rate of 48.48% (**Supplementary Table S1**). This is consistent with the conclusion from previous studies that serological testing has limited diagnostic performance in endemic areas, and thus cannot be used alone as a confirmatory diagnostic criterion for SI (Celik et al., 2012; Wang et al., 2025).

In recent years, molecular diagnostic technologies centered on NGS have delivered revolutionary breakthroughs to the diagnosis and treatment of infectious diseases. Among these, mNGS has overcome the limitations of routine bacterial culture via its broad-spectrum pathogen coverage, yet it has inherent shortcomings including high detection cost, complex workflow, long report turnaround time, and poor feasibility for routine clinical popularization, rendering it unable to serve as a conventional tool for the early screening and diagnosis of SI (Kuang et al., 2024; Zhao et al., 2024).

In contrast, tNGS is more closely aligned with the clinical needs of early SI diagnosis and management. It can specifically cover hundreds of pathogens associated with orthopedic infections. While maintaining high diagnostic sensitivity, it significantly reduces detection costs and shortens report turnaround time (Huang et al., 2024; Ding et al., 2025). In a comparative analysis enrolling 115 patients with suspected pulmonary infection, Chen et al. (2025) found that both mNGS and tNGS achieved a sensitivity of 95.08% for the diagnosis of lower respiratory tract fungal infections, with specificities of 90.74% and 85.19%, respectively. The two methods had comparable diagnostic performance, both of which were significantly superior to conventional microbiological testing, with higher detection rates for common fungi and mixed infections, and can thus serve as an effective supplementary approach to routine testing. However, prior to this study, there remained a lack of high-quality evidence-based data from large-sample multicenter studies on the systematic application value and clinical positioning of tNGS in the early diagnosis of SI.

The results of this study demonstrated that tNGS achieved an overall pathogen positive rate as high as 87.23%, and maintained stable and excellent detection performance across the three major SI subtypes (STB, BS, and PS), with positive rates reaching 84.38%, 87.88%, and 89.66%, respectively. This reflects its broad-spectrum and stable etiological diagnostic capability, which effectively addresses the key limitations of previous detection methods, including low positive rates and wide variability in detection performance across different disease subtypes.

To address the key clinical challenge of non-specific pathological changes in early SI, among the 46 cases in this study where histopathology only indicated non-specific inflammatory cell infiltration and failed to confirm the etiology, tNGS successfully detected pathogenic pathogens in 91.30% of cases, with a 100% diagnostic rate in BS cases. After combining with routine bacterial culture and Xpert MTB/RIF assay, the overall diagnostic rate reached 95.65% with a positive result from any of the etiological methods, and only 2 cases were retrospectively diagnosed by the final comprehensive clinical diagnosis. These findings provide direct etiological evidence for targeted antimicrobial therapy in early SI.

The agreement analysis showed that the Cohen’s kappa coefficients between tNGS and Xpert MTB/RIF assay, as well as between tNGS and conventional routine bacterial culture, ranged from 0.411 to 0.556 (95% CIs: 0.209–0.738), indicating moderate agreement. This level of concordance reflects the incremental sensitivity of tNGS rather than bidirectional contradictory results: across all three comparisons, discordant cases were predominantly tNGS-positive/traditional-method-negative, and the final comprehensive clinical diagnosis confirmed these as true infections missed by conventional assays. Consequently, tNGS should not be viewed as a replacement for traditional detection methods, but as a clinically complementary component that enhances the overall diagnostic yield of the multimodal strategy.

Several limitations of this study should be acknowledged. First, the retrospective design inherently carries risks of selection bias and information bias, as patient enrollment was dependent on clinical decision-making and electronic medical record completeness rather than prospective protocol-driven screening; consequently, the causal relationship between the puncture approach or tNGS-based diagnosis and clinical outcomes cannot be firmly established. Second, the single-operator-per-center design, while ensuring procedural standardization, introduces operator-dependent bias and limits the assessment of inter-operator reproducibility. The observed learning curve gradient in fluoroscopy exposure across centers suggests that broader generalization will require structured training and prospective validation involving multiple operators with varying levels of experience. Third, the study focused on perioperative procedural performance and immediate diagnostic yield, with no long-term follow-up data available; therefore, the long-term efficacy of pathogen-directed therapy, fusion outcomes, recurrence rates, and late-onset complications remain unknown. Fourth, all patients were enrolled from three centers in Northwest China, a region with a distinctive epidemiological profile characterized by a high prevalence of brucellosis and spinal tuberculosis. This regional pathogen spectrum may not be representative of other geographic areas, and the generalizability of our findings to populations with different etiological distributions should be interpreted with caution. Future prospective, nationwide multicenter studies with multiple operators per center and extended follow-up are warranted to validate these preliminary findings and optimize the broader applicability of this diagnostic strategy. Fifth, conventional routine bacterial culture was performed using standard aerobic and anaerobic media with a 14-day incubation period, without routine use of Lowenstein-Jensen medium or BACTEC MGIT systems. This suboptimal mycobacterial culture capacity, consistent with standard orthopedic clinical microbiology laboratories, precluded a true gold-standard comparison for spinal tuberculosis cases and may have contributed to the apparent superiority of molecular methods in this subtype.

## 5 Conclusion

The percutaneous paramedian small-angle lateral intervertebral foramen Kambin’s triangle approach for lumbar puncture biopsy, innovatively developed in this study, enabled acquisition of lesion specimens with acceptable safety and feasibility in this retrospective cohort under standardized training, and addressed the key clinical challenge of specimen acquisition for early etiological diagnosis of SI. Prospective validation across multiple operators is warranted before widespread adoption.

The multimodal etiological diagnostic system combining tNGS and routine bacterial culture established based on this technique achieved an overall positive rate of 91.49%, maintained stable and high detection performance across all SI subtypes, and effectively addressed the inherent limitation of traditional detection methods in early diagnosis. In summary, the diagnostic strategy of minimally invasive precise puncture combined with tNGS-based etiological testing can serve as the core strategy for early precision diagnosis and management of SI, providing critical evidence-based support for clinical targeted antimicrobial therapy.

## Data Availability

The raw data supporting the conclusions of this article will be made available by the authors, without undue reservation.
