# Supplementary material for: Diagnostic value of percutaneous paramedian small-angle lateral intervertebral foramen Kambin’s triangle approach for lumbar puncture biopsy combined with tNGS-based multimodal etiological diagnosis in early spinal infection: a multicenter retrospective diagnostic yield study
Source: Front Cell Infect Microbiol. 2026 Jul 15;16:1865471. doi: 10.3389/fcimb.2026.1865471 (PMC13414123; doi:10.3389/fcimb.2026.1865471)
Supplement: Supplementary file 1 [file DataSheet1.docx]

Between-group comparability analysis showed that there were no statistically significant differences in age, underlying comorbidities, epidemiological exposure history, previous history of tuberculosis, Visual Analogue Scale (VAS) score for low back pain, incidence of fever, etiological composition, preoperative laboratory inflammatory and etiological biomarkers, or preoperative imaging features among the three groups (all *P* > 0.05), with only a statistically significant difference in gender distribution between groups (P=0.0010). Detailed data are presented in Supplementary Tables 1 to 2.

Regarding the overall cohort characteristics, patients had varying degrees of elevation in preoperative inflammatory biomarkers, including routine blood test parameters, erythrocyte sedimentation rate (ESR), C-reactive protein (CRP), procalcitonin (PCT), and interleukin-6 (IL-6). The positive rates of traditional non-invasive etiological examinations were generally low: the overall positive rate of blood culture was 8.51%; the overall positive rate of T-SPOT.TB was 44.68%, with a positive rate of only 65.63% among the 32 patients with a final confirmed diagnosis of STB; the overall positive rate of Brucella SAT was 20.21% (19/94), with a positive rate of only 51.52% among the 33 patients with a final confirmed diagnosis of BS. These findings indicated that traditional non-invasive etiological examinations had limited diagnostic performance and a high false-negative rate in SI.

For imaging findings, the most common lesion segments were L3-4 (32 cases, 34.04%) and L4-5 (34 cases, 36.17%), which together accounted for 70.21% of the total cases. On MRI T2-weighted imaging, 88 patients (93.62%) presented with increased signal intensity of the intervertebral disc, a typical inflammatory manifestation. Vertebral bone marrow edema was focal in 50 cases (53.19%) and diffuse in 44 cases (46.81%). The overall incidence of paravertebral abscess was 48.94%, and the overall incidence of epidural abscess was 20.21%.

Supplementary Table 1. Comparison of Preoperative Laboratory Findings Across 3 Centers

| **Index** | **Center A**  **(n=43)** | **Center B**  **(n=30)** | **Center C**  **(n=21)** | **P-value** |
| --- | --- | --- | --- | --- |
| ESR (mm/h) | 39.06±27.69 | 55.73±35.12 | 45.67±32.90 | 0.0880 |
| CRP (mg/L) | 56.74±56.69 | 52.50±42.95 | 52.32±51.19 | 0.9212 |
| PCT (ng/mL) | 0.12±0.14 | 0.10±0.12 | 0.14±0.25 | 0.7666 |
| IL-6 (pg/mL) | 29.20±21.59 | 37.70±37.51 | 22.55±18.48 | 0.1414 |
| WBC Count (×10^9/L) | 6.63±4.11 | 7.18±4.82 | 6.46±2.93 | 0.7936 |
| Neutrophil (%), % | 64.02±13.20 | 66.88±12.24 | 61.11±12.51 | 0.2830 |
| Monocyte (%), % | 9.12±2.44 | 8.77±2.52 | 9.18±2.66 | 0.8024 |
| Lymphocyte (%), % | 24.84±11.73 | 22.42±10.75 | 26.97±12.15 | 0.3764 |
| Positive Blood Culture, n (%) | 3 (6.98) | 3 (10.00) | 2 (9.52) | 0.8856 |
| Positive T-SPOT.TB, n (%) | 20 (46.51) | 13 (43.33) | 9 (42.86) | 0.9471 |
| Positive Brucella SAT, n (%) | 11 (25.58) | 4 (13.33) | 4(19.05) | 0.4346 |

Supplementary Table 2. Comparison of Preoperative Imaging Characteristics Across 3 Centers

| **Imaging Index** | **Center A**  **(n=43)** | **Center B**  **(n=30)** | **Center C**  **(n=21)** | **P-value** |
| --- | --- | --- | --- | --- |
| Puncture Lesion Disc Distribution Segments, n (%) |  |  |  |  |
| - L1-2 | 4 (9.30) | 1 (3.33) | 1 (4.76) | 0.5564 |
| - L2-3 | 7 (16.28) | 5 (16.67) | 3 (14.29) | 0.9713 |
| - L3-4 | 12 (27.91) | 12 (40.00) | 8 (38.10) | 0.5095 |
| - L4-5 | 18 (41.86) | 9 (30.00) | 7 (33.33) | 0.5568 |
| - L5-S1 | 2 (4.65) | 3 (10.00) | 2 (9.52) | 0.6367 |
| Disc Signal, n (%) |  |  |  |  |
| - Hypointense | 2 (4.65) | 1 (3.33) | 1 (4.76) | 0.8928 |
| - No Significant Signal Change | 2 (4.65) | 1 (3.33) | 0 (0.00) |  |
| - Hyperintense | 39 (90.70) | 29 (96.67) | 20 (95.24) |  |
| Vertebral Marrow Edema, n (%) |  |  |  |  |
| - Focal (<1/2 Vertebra) | 23 (53.49) | 13 (43.33) | 14 (66.67) | 0.2587 |
| - Diffuse (≥1/2 Vertebra) | 20 (46.51) | 17 (56.67) | 7 (33.33) |  |
| Paraspinal Abscess, n (%) |  |  |  |  |
| - Unilateral | 15 (34.88) | 10 (33.33) | 5 (23.81) | 0.3758 |
| - Bilateral | 10 (23.26) | 4 (13.33) | 2 (9.52) |  |
| - No Paraspinal Abscess | 18 (41.86) | 16 (53.33) | 14 (66.67) |  |
| Epidural Abscess, n (%) | 9 (20.93) | 6 (20.00) | 4 (19.05) | 0.9840 |
